# Supplementary material for: Mid-infrared group IV nanowire laser
Source: Sci Adv. 2025 May 16;11(20):eadt6723. doi: 10.1126/sciadv.adt6723 (PMC12083537; doi:10.1126/sciadv.adt6723)
Supplement: Supplementary file 1 — Supplementary Text Figs. S1 to S7 Table S1 References [file sciadv.adt6723_sm.pdf]

Science Advances
AAAS

Supplementary Materials for

Mid-infrared group IV nanowire laser

Youngmin Kim *et al.*

Corresponding author: Oussama Moutanabbir, oussama.moutanabbir@polymtl.ca;
Donguk Nam, dwnam@kaist.ac.kr

Sci. Adv. **11**, eadt6723 (2025)
DOI: 10.1126/sciadv.adt6723

This PDF file includes:

Supplementary Text
Figs. S1 to S7
Table S1
References

Supplementary Text

Calculation of strain profiles of a nanowire before and after strain engineering

To estimate the extent of relaxed compressive strain in the nanowire through strain engineering, we performed strain simulations using the finite-element method (FEM). This approach is in line with the simulation methodology described in Albani *et al.* (*41*) and Assali *et al.* (*42*), which offers an accurate delineation of the strain fields within the realistic three-dimensional configuration of the nanowire. The lattice mismatch between the Ge core and GeSn shell is set to the initial compressive strain of the GeSn shell, while the Ge core is assumed to be initially undeformed. The strain profile is then determined by solving the mechanical equilibrium in the inhomogeneous system. The lattice mismatch-induced strain (ε) is represented as:

$$\varepsilon = \frac{a_{Ge} - a_{GeSn}}{a_{GeSn}} \qquad (S1)$$

where a_{GeSn} and a_{Ge} signify the lattice constants of GeSn and Ge, respectively, with a_{Ge} possessing a value of 5.657 Å (*41*). The lattice constant of GeSn (a_{GeSn}) as a function of Sn composition is given by:

$$a_{GeSn} = a_{Ge}(1-x) + a_{Sn}x + b_{GeSn}(1-x) \qquad (S2)$$

in which a_{sn} and b_{GeSn} denote the lattice constant of Sn and bowing parameter, respectively, with values of 6.489 Å and 0.041 Å (*41, 43*). For the strain-engineered nanowires, the simulation incorporates the application of a 207 nm thick SiO_2 layer imposing a compressive stress of 470 MPa, consistent with the experimentally measured stress in the SiO_2 stressor. Our analysis primarily focused on axial strain due to its significant variation (~0.3%) after strain engineering, in contrast to the negligible changes (<0.1%) revealed in the radial and tangential components. Fig. S1A presents a simulated cross-sectional <111> axial strain profile of a nanowire prior to strain engineering, showcasing the compressive strain within the GeSn shell due to the inherent lattice mismatch between the Ge core and GeSn shell. This compressive strain within GeSn shell is estimated to average around –0.3%. Building on the findings of Assali *et al.* (*42*), which illustrated a notable reduction in compressive strain from –0.9% to –0.3% with the reduction of the Ge core diameter from 100 nm to 20 nm, our simulations mirrored this trend. By employing a thin Ge core with 20 nm diameter, we attained a similarly low residual compressive strain of –0.3% in the GeSn shell, thus validating the consistency of our approach with established research. Despite this low residual strain, detrimental strain persisted in the GeSn shell, necessitating mitigation through the application of a SiO_2 stressor. Fig. S1B shows a simulated cross-sectional axial strain profile of a nanowire after SiO_2 stressor deposition, which evidently alleviates the compressive strain in the GeSn shell due to the expansion tendency of the SiO_2 stressor layer. The average strain in the GeSn shell is calculated to be ~0.0%. Based on these findings, we estimated the compressive strain relaxation in the GeSn shell to be approximately 0.3% along the <111> axial direction of the nanowire.

Raman analysis of nanowires before and after SiO_x stressor deposition

To confirm that the stressor deposition process does not induce damage to the nanowires, we performed the Raman spectroscopy measurement, which is effective in assessing defects and disorders in materials. Fig. S2 shows the measured Raman spectra with Lorentzian fitting of the nanowires before (red curve) and after (yellow curve) SiO_x stressor deposition. The full-width at half-maximum (FWHM) of the Raman spectra for the nanowires before and after stressor deposition show comparable values of 5.8 and 6.1 cm^{-1}, respectively. This consistency confirms that the nanowires remain undamaged following SiO_x stressor deposition. Additionally, the Raman peak positions of the nanowires are observed at 292.2 cm^{-1} before deposition and 290.2 cm^{-1} after deposition. While the peak positions are clearly defined, an accurate estimation of strain is impossible due to the absence of a reported Raman <111> strain-shift coefficient for GeSn with a Sn content of ~10 at.%.

3D FDTD analysis of nanowires with varied sidewall angles

To investigate the impact of facet sidewall angle deviations resulting from nonideal FIB milling on optical confinement, we performed additional three-dimensional (3D) finite-difference time-domain (FDTD) simulations. The materials employed for the simulations included GeSn, Ge, SiO_2, and Si with refractive indices of 4.3, 4.1, 1.44, and 3.4, respectively. The nanowires were modeled with a length of 17 μm, a GeSn shell diameter of 480 nm, and a Ge core diameter of 20 nm. The simulations focused on a wavelength of approximately 2300 nm, corresponding to the peak of our lasing spectrum. Q factors were determined by analyzing the slope of the electromagnetic field intensity decay over several round trips of light within the nanowire cavity. Notably, the optical mode profile from the simulations is identified as HE11 mode, which is generally observed in the nanowire geometry (*8*).

Fig. S3 shows the Q factors as a function of nanowire facet sidewall angle, calculated by 3D FDTD simulations. The sidewall angle varies from 0° to 10° relative to the vertical axis, a range deemed feasible based on the sharply etched nanowire facets observed in the top-view SEM image (Fig. 1B, bottom right). Nanowires with sidewall angles from 1° to 10° show Q factors ranging from 213 to 217, which is a slight deviation of Q factor < 4 compared to nanowires with a perfectly vertical angle of 0° (Q ~217). This confirms that slight deviations from perfectly vertical sidewalls do not significantly compromise optical confinement.

Theoretical modeling of the gain and loss

The calculations of interband gain and inter-valence band absorption were made using the 8-band **k·p** model, described in detail in Chuang *et al.* (*44*). The Luttinger parameters for the GeSn alloy were taken from Liu *et al.* (*45*), and other parameters are given in Rainko *et al.* (*46*). To find the net gain, free-carrier absorption can be evaluated using the second-order perturbation model described in detail by Tsai *et al.* (*47*), which incorporates various scattering mechanisms including acoustic phonon, deformation potential in the L-valley, intervalley, ionized impurity, and alloy scattering. An alternative method involves applying empirical expressions fitted to experimental data for germanium (*48, 49*), which we opted for in this analysis. Also, we accounted for indirect absorption—potentially significant in group-IV materials and directed towards the L- and X-valleys in the conduction band—as elucidated by Virgilio *et al.* (*50*) and Trupke *et al.* (*51*). However, in the structures considered here its impact was found to be very small.

Calculation of threshold gains of a nanowire before and after cavity optimization

The estimation of the threshold gain is derived from the threshold modal gain equation (*52*). The equation is expressed as follows:

$$\Gamma g_{th} = k_0 n_g / Q \qquad (S3)$$

where Γ represents the confinement factor, Γg_{th} denotes the threshold modal gain, n_g is the group index, k_0 symbolizes the angular wavenumber, and Q is the quality factor of the nanowire cavity. The expression for k_0 is given by $k_0 = 2\pi/\lambda$, where λ is the wavelength of the emitted light. To ascertain the values of the involved parameters, finite-difference time-domain (FDTD) simulations were employed. Through the simulations, the confinement factor (Γ) and group index (n_g) were deduced to 0.60 and ~5.7, respectively. Moreover, the Q factors of the nanowire prior to and post cavity optimization were calculated to be 183 and 217, respectively. Consequently, the threshold gains (g_{th}) of the nanowire before and after cavity optimization are estimated to be 1418 and 1196 cm^{-1}, respectively.

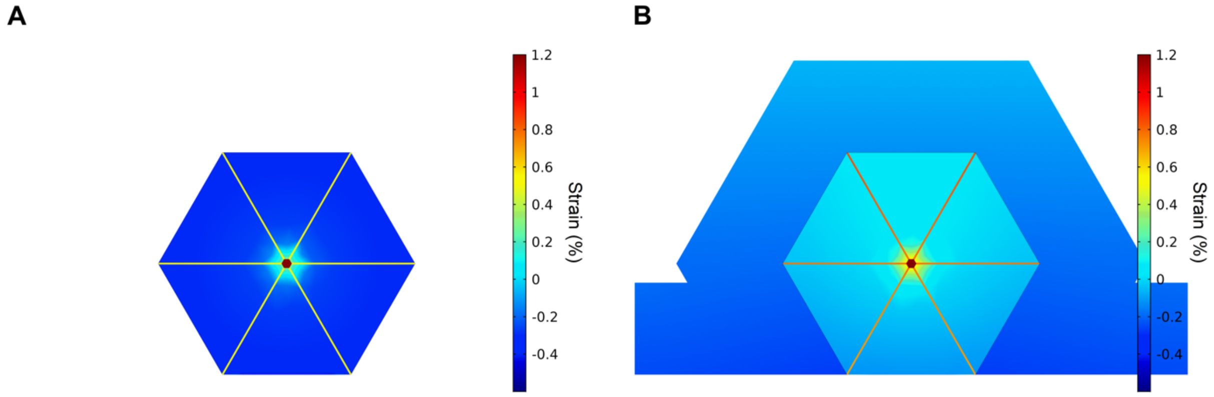

Fig. S1. Simulated strain profiles of a nanowire before and after strain engineering. (**A**) Cross-sectional axial strain profile of a nanowire prior to strain engineering, showing the presence of residual compressive strain in the GeSn shell attributed to the growth-induced lattice mismatch between Ge core and GeSn shell. The compressive strain in the GeSn shell is calculated to average around –0.3%. (**B**) Cross-sectional axial strain profile of a nanowire with an SiO_2 stressor deposition, revealing a relaxation of compressive strain in the GeSn shell. The average strain in the GeSn shell is calculated to be approximately 0.0%.

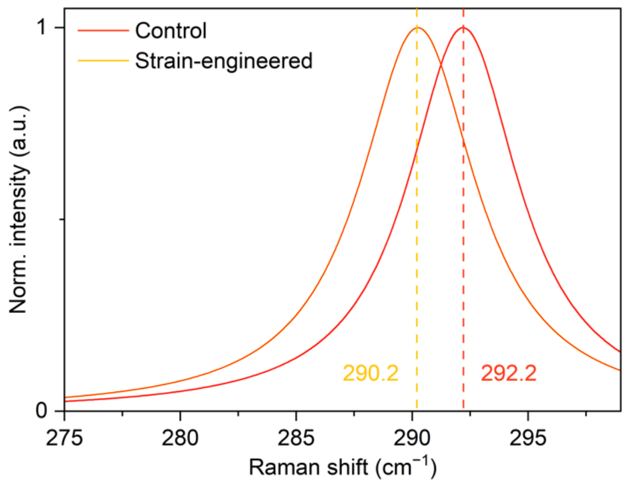

Fig. S2. Raman spectra of control and strain-engineered nanowires with Lorentzian fitting. The control and strain-engineered nanowires exhibit Raman peak positions at 292.2 and 290.2 cm^{-1}, respectively. The FWHM of the Raman spectra for control and strain-engineered nanowires are 5.8 and 6.1 cm^{-1}, respectively, indicating that the nanowires remain undamaged following SiO_x stressor deposition.

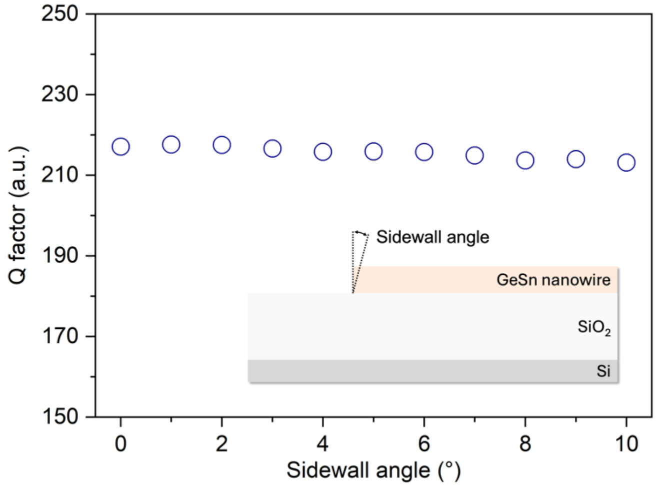

Fig. S3. Q factors as a function of nanowire facet sidewall angle. The sidewall angle varies from 0° to 10° relative to the vertical axis, a feasible range based on the very sharply etched nanowire facets observed in the top-view SEM image (Fig. 1B, bottom right). The nanowires with sidewall angles from 1° to 10° show slight deviations of Q factors < 4 compared to the nanowire with a perfectly vertical angle of 0°, confirming that slight deviations from perfectly vertical sidewalls do not significantly compromise optical confinement.

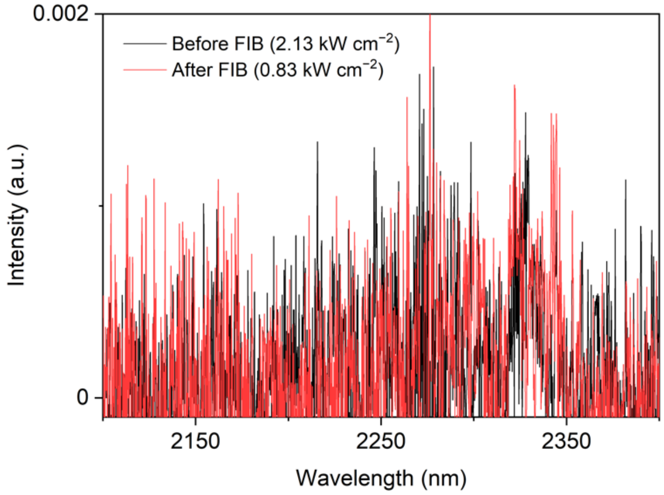

Fig. S4. Photoluminescence emission spectra of the GeSn nanowires before and after FIB milling. The nanowires are pumped with power densities of 2.13 and 0.83 kW cm^{-2} before and after FIB milling, respectively, both below the lasing threshold. Even with the lower pump power after FIB, the emissions remain comparable to those before FIB, indicating that the overall optical properties of the nanowires are largely preserved. This suggests that the optical performance of the nanowires is negligibly affected by the FIB milling process.

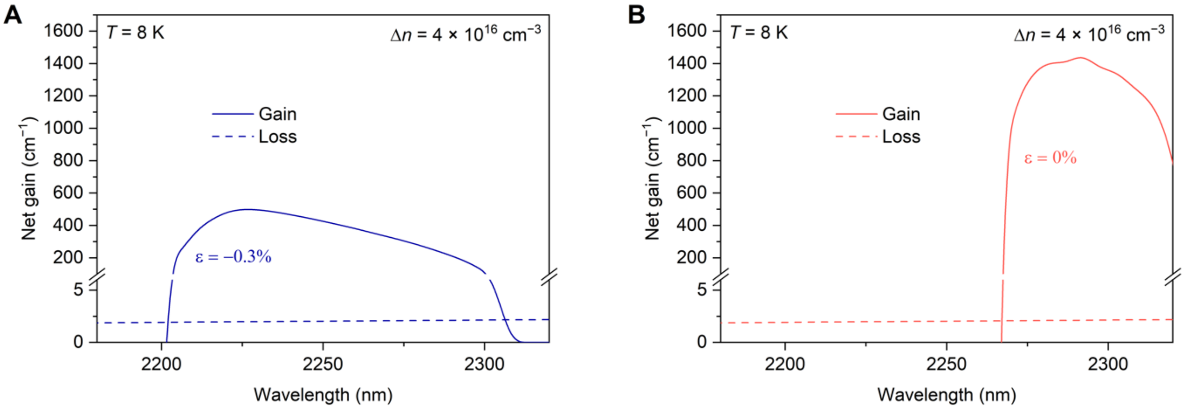

Fig. S5. Theoretical modeling for material gain and loss in a strain-engineered GeSn nanowire at 8 K. Calculated gain (solid lines) loss (dashed line) at an injection density of 4 × 10^{16} cm^{-3} for the nanowire under strains of –0.3% (blue curves, **A**) and 0% (blue curves, **B**), which correspond to the strains in non-strain-engineered and strain-engineered nanowires, respectively. The loss considers all possible loss mechanisms including IVBA, FCA, and Γ–L transfer, but the dominant mechanism limiting material gain is determined to be FCA due to the relatively low injected carrier density. The overall material loss also remains small due to low injected carrier density. The net gain, shown in Fig. 3, is determined by subtracting the loss from the gain for each strain.

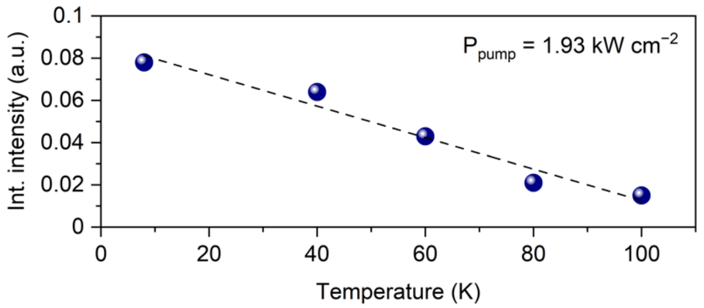

Fig. S6. Integrated photoluminescence intensity as a function of temperature. A pump power density is fixed at 1.93 kW cm^{-2}, which is below the lasing threshold. The integrated photoluminescence intensity exhibits a decrease in a nearly linear manner across the temperature range, including at 100 K where lasing quenches.

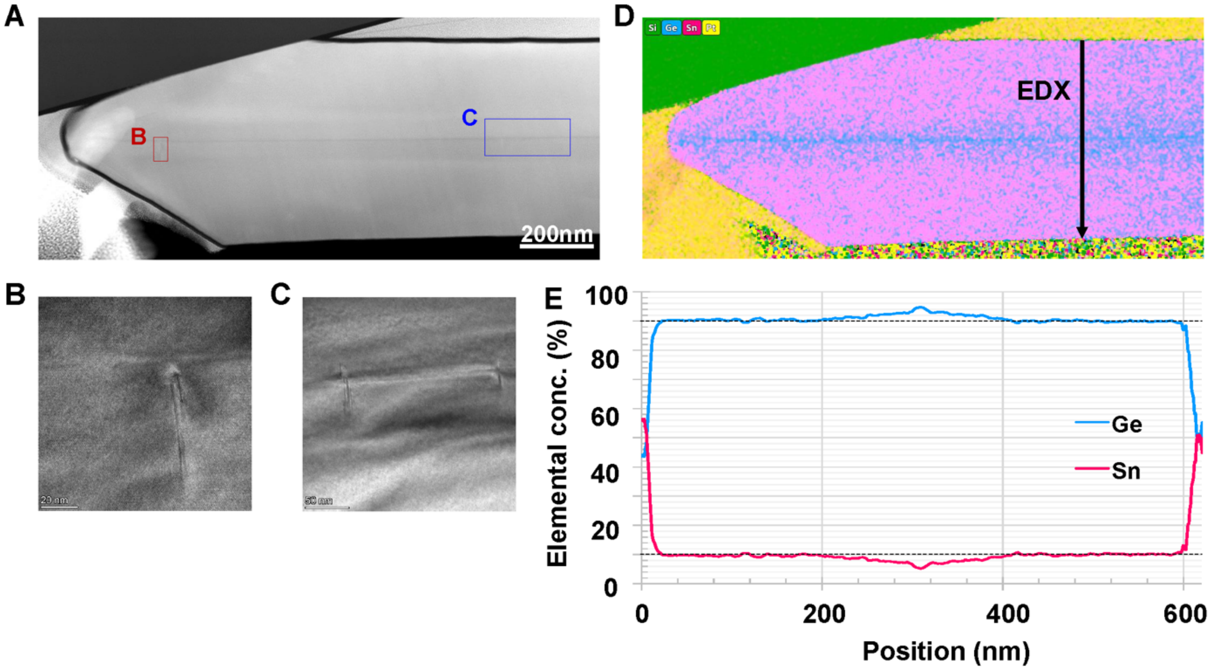

Fig. S7. Transmission electron microscopy (TEM) analysis of a Ge/GeSn core/shell nanowire. TEM images showing a FIB cut section along the growth axis (**A**). The TEM lamella is approximately 50 nm thick. The nanowires have a diameter of about 500nm with a 240nm GeSn-shell grown around a 20nm Ge core. In the 2 µm imaged along the growth axis, 3 defects are found (**B**, **C**). All 3 defects are dislocations only visible close to the core/shell interface. Note, that energy dispersive X-ray (EDX) analysis data (**D**, **E**) indicates that in agreement with other studies (*28*), the Sn concentration near the core is lower and hence the bandgap is larger than in the outer parts of the shell. As a result, the dislocations are likely to be spatially separated from the carriers.

Table S1. Lasing threshold comparison. The threshold power density of the GeSn nanowire laser is lower than that observed in relaxed GeSn lasers with a comparable Sn content fabricated on a thin-film platform.

Ref.	Sn content (%)	Strain (%)	Threshold ($kW\ cm^{-2}$)	Temperature (K)	Cavity	Q factor
(*19*)	10.5	0%	11.6	25	Microdisk	-
(*21*)	10.4	0%	17	4	Microdisk	15000
(*20*)	10.6	0%	18.2	4	Photonic crystal	14600
(*25*)	10.6	0%	50.4	4	Corner-cube	480
This work	~10	0%	5.3	8	Nanowire	217

REFERENCES AND NOTES

1. M. E. Reimer, G. Bulgarini, N. Akopian, M. Hocevar, M. B. Bavinck, M. A. Verheijen, E. P. A. M. Bakkers, L. P. Kouwenhoven, V. Zwiller, Bright single-photon sources in bottom-up tailored nanowires. *Nat. Commun.* **3**, 737 (2012).

2. S. W. Eaton, A. Fu, A. B. Wong, C.-Z. Ning, P. Yang, Semiconductor nanowire lasers. *Nat. Rev. Mater.* **1**, 16028 (2016).

3. R. Yan, D. Gargas, P. Yang, Nanowire photonics. *Nat. Photon.* **3**, 569–576 (2009).

4. M. H. Huang, S. Mao, H. Feick, H. Yan, Y. Wu, H. Kind, E. Weber, R. Russo, P. Yang, Room-temperature ultraviolet nanowire nanolasers. *Science* **292**, 1897–1899 (2001).

5. J. C. Johnson, H.-J. Choi, K. P. Knutsen, R. D. Schaller, P. Yang, R. J. Saykally, Single gallium nitride nanowire lasers. *Nat. Mater.* **1**, 106–110 (2002).

6. X. Duan, Y. Huang, R. Agarwal, C. M. Lieber, Single-nanowire electrically driven lasers. *Nature* **421**, 241–245 (2003).

7. F. Qian, Y. Li, S. Gradečak, H.-G. Park, Y. Dong, Y. Ding, Z. L. Wang, C. M. Lieber, Multi-quantum-well nanowire heterostructures for wavelength-controlled lasers. *Nat. Mater.* **7**, 701–706 (2008).

8. D. Saxena, S. Mokkapati, P. Parkinson, N. Jiang, Q. Gao, H. H. Tan, C. Jagadish, Optically pumped room-temperature GaAs nanowire lasers. *Nat. Photon.* **7**, 963–968 (2013).

9. H. Sumikura, G. Zhang, M. Takiguchi, N. Takemura, A. Shinya, H. Gotoh, M. Notomi, Mid-infrared lasing of single wurtzite InAs nanowire. *Nano Lett.* **19**, 8059–8065 (2019).

10. Q. Bao, W. Li, P. Xu, M. Zhang, D. Dai, P. Wang, X. Guo, L. Tong, On-chip single-mode CdS nanowire laser. *Light Sci. Appl.* **9**, 42 (2020).

11. Z. Zhou, B. Yin, J. Michel, On-chip light sources for silicon photonics. *Light Sci. Appl.* **4**, e358 (2015).

12. O. Moutanabbir, S. Assali, X. Gong, E. O'Reilly, C. A. Broderick, B. Marzban, J. Witzens, W. Du, S. Q. Yu, A. Chelnokov, D. Buca, D. Nam, Monolithic infrared silicon photonics: The rise of (Si)GeSn semiconductors. *Appl. Phys. Lett.* **118**, 110502 (2021).

13. L. M. Rosenfeld, D. A. Sulway, G. F. Sinclair, V. Anant, M. G. Thompson, J. G. Rarity, J. W. Silverstone, Mid-infrared quantum optics in silicon. *Opt. Express* **28**, 37092–37102 (2020).

14. E. M. T. Fadaly, A. Dijkstra, J. R. Suckert, D. Ziss, M. A. J. van Tilburg, C. Mao, Y. Ren, V. T. van Lange, K. Korzun, S. Kölling, M. A. Verheijen, D. Busse, C. Rödl, J. Furthmüller, F. Bechstedt, J. Stangl, J. J. Finley, S. Botti, J. E. M. Haverkort, E. P. A. M. Bakkers, Direct-bandgap emission from hexagonal Ge and SiGe alloys. *Nature* **580**, 205–209 (2020).

15. M. A. J. van Tilburg, R. Farina, V. T. van Lange, W. H. J. Peeters, S. Meder, M. M. Jansen, M. A. Verheijen, M. Vettori, J. J. Finley, E. P. A. M. Bakkers, J. E. M. Haverkort, Stimulated emission from hexagonal silicon-germanium nanowires. *Commun. Phys.* **7**, 328 (2024).

16. S. Gupta, B. Magyari-Köpe, Y. Nishi, K. C. Saraswat, Achieving direct band gap in germanium through integration of Sn alloying and external strain. *J. Appl. Phys.* **113**, 073707 (2013).

17. S. Wirths, R. Geiger, N. von den Driesch, G. Mussler, T. Stoica, S. Mantl, Z. Ikonic, M. Luysberg, S. Chiussi, J. M. Hartmann, H. Sigg, J. Faist, D. Buca, D. Grützmacher, Lasing in direct-bandgap GeSn alloy grown on Si. *Nat. Photon.* **9**, 88–92 (2015).

18. A. Elbaz, D. Buca, N. von den Driesch, K. Pantzas, G. Patriarche, N. Zerounian, E. Herth, X. Checoury, S. Sauvage, I. Sagnes, A. Foti, R. Ossikovski, J.-M. Hartmann, F. Boeuf, Z. Ikonic, P. Boucaud, D. Grützmacher, M. El Kurdi, Ultra-low-threshold continuous-wave and pulsed lasing in tensile-strained GeSn alloys. *Nat. Photon.* **14**, 375–382 (2020).

19. A. Elbaz, R. Arefin, E. Sakat, B. Wang, E. Herth, G. Patriarche, A. Foti, R. Ossikovski, S. Sauvage, X. Checoury, K. Pantzas, I. Sagnes, J. Chrétien, L. Casiez, M. Bertrand, V. Calvo, N. Pauc, A. Chelnokov, P. Boucaud, F. Boeuf, V. Reboud, J.-M. Hartmann, M. El Kurdi, Reduced lasing thresholds in GeSn microdisk cavities with defect management of the optically active region. *ACS Photon.* **7**, 2713–2722 (2020).

20. H.-J. Joo, Y. Kim, D. Burt, Y. Jung, L. Zhang, M. Chen, S. J. Parluhutan, D.-H. Kang, C. Lee, S. Assali, Z. Ikonic, O. Moutanabbir, Y.-H. Cho, C. S. Tan, D. Nam, 1D photonic crystal direct bandgap GeSn-on-insulator laser. *Appl. Phys. Lett.* **119**, 201101 (2021).

21. Y. Jung, D. Burt, L. Zhang, Y. Kim, H.-J. Joo, M. Chen, S. Assali, O. Moutanabbir, C. Seng Tan, D. Nam, Optically pumped low-threshold microdisk lasers on a GeSn-on-insulator substrate with reduced defect density. *Photon. Res.* **10**, 1332–1337 (2022).

22. Y. Kim, S. Assali, D. Burt, Y. Jung, H.-J. Joo, M. Chen, Z. Ikonic, O. Moutanabbir, D. Nam, Enhanced GeSn microdisk lasers directly released on Si. *Adv. Opt. Mater.* **10**, 2101213 (2022).

23. H.-J. Joo, Y. Kim, M. Chen, D. Burt, L. Zhang, B. Son, M. Luo, Z. Ikonic, C. Lee, Y.-H. Cho, C. S. Tan, D. Nam, All-around HfO_2 stressor for tensile strain in GeSn-on-insulator nanobeam lasers. *Adv. Opt. Mater.* **11**, 2301115 (2023).

24. Y. Kim, H.-J. Joo, M. Chen, B. Son, D. Burt, X. Shi, L. Zhang, Z. Ikonic, C. S. Tan, D. Nam, High-precision wavelength tuning of GeSn nanobeam lasers via dynamically controlled strain engineering. *Adv. Sci.* **10**, e2207611 (2023).

25. H.-J. Joo, J. Liu, M. Chen, D. Burt, B. Chomet, Y. Kim, X. Shi, K. Lu, L. Zhang, Z. Ikonic, Y.-I. Sohn, C. S. Tan, D. Gacemi, A. Vasanelli, C. Sirtori, Y. Todorov, D. Nam, Actively tunable laser action in GeSn nanomechanical oscillators. *Nat. Nanotechnol.* **19**, 1116–1121 (2024).

26. S. Biswas, J. Doherty, D. Saladukha, Q. Ramasse, D. Majumdar, M. Upmanyu, A. Singha, T. Ochalski, M. A. Morris, J. D. Holmes, Non-equilibrium induction of tin in germanium: Towards direct bandgap $Ge_{1-x}Sn_x$ nanowires. *Nat. Commun.* **7**, 11405 (2016).

27. A. C. Meng, C. S. Fenrich, M. R. Braun, J. P. McVittie, A. F. Marshall, J. S. Harris, P. C. McIntyre, Core-shell germanium/germanium–tin nanowires exhibiting room-temperature direct- and indirect-gap photoluminescence. *Nano Lett.* **16**, 7521–7529 (2016).

28. S. Assali, A. Dijkstra, A. Li, S. Koelling, M. A. Verheijen, L. Gagliano, N. von den Driesch, D. Buca, P. M. Koenraad, J. E. M. Haverkort, E. P. A. M. Bakkers, Growth and optical properties of direct band gap Ge/$Ge_{0.87}Sn_{0.13}$ core/shell nanowire arrays. *Nano Lett.* **17**, 1538–1544 (2017).

29. M. S. Seifner, A. Dijkstra, J. Bernardi, A. Steiger-Thirsfeld, M. Sistani, A. Lugstein, J. E. M. Haverkort, S. Barth, Epitaxial $Ge_{0.81}Sn_{0.19}$ nanowires for nanoscale mid-infrared emitters. *ACS Nano* **13**, 8047–8054 (2019).

30. A. C. Meng, M. R. Braun, Y. Wang, C. S. Fenrich, M. Xue, D. R. Diercks, B. P. Gorman, M. I. Richard, A. F. Marshall, W. Cai, J. S. Harris, P. C. McIntyre, Coupling of coherent misfit strain and composition distributions in core–Shell Ge/$Ge_{1-x}Sn_x$ nanowire light emitters. *Mater. Today Nano* **5**, 100026 (2019).

31. S. Assali, R. Bergamaschini, E. Scalise, M. A. Verheijen, M. Albani, A. Dijkstra, A. Li, S. Koelling, E. P. A. M. Bakkers, F. Montalenti, L. Miglio, Kinetic control of morphology and composition in Ge/GeSn core/shell nanowires. *ACS Nano* **14**, 2445–2455 (2020).

32. A. C. Meng, M. R. Braun, Y. Wang, S. Peng, W. Tan, J. Z. Lentz, M. Xue, A. Pakzad, A. F. Marshall, J. S. Harris, W. Cai, P. C. McIntyre, Growth mode control for direct-gap core/shell Ge/GeSn nanowire light emission. *Mater. Today* **40**, 101–113 (2020).

33. Y. Kim, S. Assali, H.-J. Joo, S. Koelling, M. Chen, L. Luo, X. Shi, D. Burt, Z. Ikonic, D. Nam, O. Moutanabbir, Short-wave infrared cavity resonances in a single GeSn nanowire. *Nat. Commun.* **14**, 4393 (2023).

34. A. Gassenq, S. Tardif, K. Guilloy, I. Duchemin, N. Pauc, J. M. Hartmann, D. Rouchon, J. Widiez, Y. M. Niquet, L. Milord, T. Zabel, H. Sigg, J. Faist, A. Chelnokov, F. Rieutord, V. Reboud, V. Calvo, Raman-strain relations in highly strained Ge: Uniaxial □100□, □110□ and biaxial (001) stress. *J. Appl. Phys.* **121**, 055702 (2017).

35. L. Luo, M. R. M. Atalla, S. Assali, S. Koelling, G. Daligou, O. Moutanabbir, Mid-infrared imaging using strain-relaxed $Ge_{1-}xSn_{x}$ alloys grown on 20 nm Ge nanowires. *Nano Lett.* **24**, 4979–4986 (2024).

36. J. Wang, M. S. Gudiksen, X. Duan, Y. Cui, C. M. Lieber, Highly polarized photoluminescence and photodetection from single indium phosphide nanowires. *Science* **293**, 1455–1457 (2001).

37. P. Yu, Z. Li, T. Wu, Y.-T. Wang, X. Tong, C.-F. Li, Z. Wang, S.-H. Wei, Y. Zhang, H. Liu, L. Fu, Y. Zhang, J. Wu, H. H. Tan, C. Jagadish, Z. M. Wang, Nanowire quantum dot surface engineering for high temperature single photon emission. *ACS Nano* **13**, 13492–13500 (2019).

38. A. Bjelajac, M. Gromovyi, E. Sakat, B. Wang, G. Patriarche, N. Pauc, V. Calvo, P. Boucaud, F. Boeuf, A. Chelnokov, V. Reboud, M. Frauenrath, J. M. Hartmann, M. El Kurdi, Up to 300 K lasing with GeSn-On-Insulator microdisk resonators. *Opt. Express* **30**, 3954–3961 (2022).

39. J. Chrétien, Q. M. Thai, M. Frauenrath, L. Casiez, A. Chelnokov, V. Reboud, J. M. Hartmann, M. El Kurdi, N. Pauc, V. Calvo, Room temperature optically pumped GeSn microdisk lasers. *Appl. Phys. Lett.* **120**, 051107 (2022).

40. D. Buca, A. Bjelajac, D. Spirito, O. Concepción, M. Gromovyi, E. Sakat, X. Lafosse, L. Ferlazzo, N. von den Driesch, Z. Ikonic, D. Grützmacher, G. Capellini, M. El Kurdi, Room temperature lasing in GeSn microdisks enabled by strain engineering. *Adv. Opt. Mater.* **10**, 2201024 (2022).

41. M. Albani, S. Assali, M. A. Verheijen, S. Koelling, R. Bergamaschini, F. Pezzoli, E. P. A. M. Bakkers, L. Miglio, Critical strain for Sn incorporation into spontaneously graded Ge/GeSn core/shell nanowires. *Nanoscale* **10**, 7250–7256 (2018).

42. S. Assali, M. Albani, R. Bergamaschini, M. A. Verheijen, A. Li, S. Kölling, L. Gagliano, E. P. A. M. Bakkers, L. Miglio, Strain engineering in Ge/GeSn core/shell nanowires. *Appl. Phys. Lett.* **115**, 113102 (2019).

43. F. Gencarelli, B. Vincent, J. Demeulemeester, A. Vantomme, A. Moussa, A. Franquet, A. Kumar, H. Bender, J. Meersschaut, W. Vandervorst, R. Loo, M. Caymax, K. Temst, M. Heyns, Crystalline properties and strain relaxation mechanism of CVD grown GeSn. *ECS J. Solid State Sci. Technol.* **2**, P134–P137 (2013).

44. S. L. Chuang, *Physics of Photonic Devices* (John Wiley & Sons, 2012).

45. S.-Q. Liu, S.-T. Yen, Extraction of eight-band k · p parameters from empirical pseudopotentials for GeSn. *J. Appl. Phys.* **125**, 245701 (2019).

46. D. Rainko, Z. Ikonic, N. Vukmirović, D. Stange, N. von den Driesch, D. Grützmacher, D. Buca, Investigation of carrier confinement in direct bandgap GeSn/SiGeSn 2D and 0D heterostructures. *Sci. Rep.* **8**, 15557 (2018).

47. T. Chin-Yi, T. Chin-Yao, C. Chih-Hsiung, S. Tien-Li, W. Tsu-Yin, S. Fang-Ping, Theoretical model for intravalley and intervalley free-carrier absorption in semiconductor lasers: Beyond the classical Drude model. *EEE J. Quantum Electron.* **34**, 552–559 (1998).

48. J. Liu, X. Sun, D. Pan, X. Wang, L. C. Kimerling, T. L. Koch, J. Michel, Tensile-strained, n-type Ge as a gain medium for monolithic laser integration on Si. *Opt. Express* **15**, 11272–11277 (2007).

49. D. Peschka, M. Thomas, A. Glitzky, R. Nürnberg, K. Gärtner, M. Virgilio, S. Guha, T. Schroeder, G. Capellini, T. Koprucki, Modeling of edge-emitting lasers based on tensile strained germanium microstrips. *IEEE Photon. J.* **7**, 1502115 (2015).

50. M. Virgilio, C. L. Manganelli, G. Grosso, G. Pizzi, G. Capellini, Radiative recombination and optical gain spectra in biaxially strained n-type germanium. *Phys. Rev. B* **87**, 235313 (2013).

51. T. Trupke, M. A. Green, P. Würfel, Optical gain in materials with indirect transitions. *J. Appl. Phys.* **93**, 9058–9061 (2003).

52. S. Bao, D. Kim, C. Onwukaeme, S. Gupta, K. Saraswat, K. H. Lee, Y. Kim, D. Min, Y. Jung, H. Qiu, H. Wang, E. A. Fitzgerald, C. S. Tan, D. Nam, Low-threshold optically pumped lasing in highly strained germanium nanowires. *Nat. Commun.* **8**, 1845 (2017).
